# Stem and leaf growth rates define the leaf size vs. number trade-off

**DOI:** 10.1093/aobpla/plz063

**Published:** 2019-11-19

**Authors:** Jun Sun, Mantang Wang, Min Lyu, Karl J Niklas, Quanlin Zhong, Man Li, Dongliang Cheng

**Affiliations:** 1 Fujian Provincial Key Laboratory of Plant Ecophysiology, Fujian Normal University, Fuzhou, Fujian Province, China; 2 School of City and Civil Engineering, Zaozhuang University, Zaozhuang, Shandong Province, China; 3 Plant Biology Section, School of Integrative Plant Science, Cornell University, Ithaca, NY, USA; 4 Institute of Geography, Fujian Normal University, Fuzhou Province, China

**Keywords:** Elevational gradient, forest communities, leafing intensity, leaf size, metabolic scaling theory, twig architecture

## Abstract

The trade-off between leaf number and individual leaf size on current-year shoots (twigs) is crucial to light interception and thus net carbon gain. However, a theoretical basis for understanding this trade-off remains elusive. Here, we argue that this trade-off emerges directly from the relationship between annual growth in leaf and stem mass, a hypothesis that predicts that maximum individual leaf size (i.e. leaf mass, *M*_max_, or leaf area, *A*_max_) will scale negatively and isometrically with leafing intensity (i.e. leaf number per unit stem mass, per unit stem volume or per stem cross-sectional area). We tested this hypothesis by analysing the twigs of 64 species inhabiting three different forest communities along an elevation gradient using standardized major axis (SMA) analyses. Across species, maximum individual leaf size (*M*_max_, *A*_max_) scaled isometrically with respect to leafing intensity; the scaling constants between maximum leaf size and leafing intensity (based on stem cross-sectional area) differed significantly among the three forests. Therefore, our hypothesis successfully predicts a scaling relationship between maximum individual leaf size and leafing intensity, and provides a general explanation for the leaf size-number trade-off as a consequence of mechanical-hydraulic constraints on stem and leaf growth per year.

## Teaser

The trade-off between leaf number and individual leaf size on current-year shoots (twigs) is crucial to light interception and thus net carbon gain. We present a model (stem-leaf growth hypothesis, SLGH) to provide a theoretical explanation for the trade-off between the maximum leaf size vs. leafing intensity. We found that the scaling exponents of maximum leaf size vs. the leafing intensity are close to −1.0 and are insensitive to forest types and different elevations. Therefore, our results successfully provide a general explanation for this trade-off as a consequence of mechanical-hydraulic constraints on stem and leaf growth per year.

## Introduction

Understanding variations of the critical functional traits of current-year shoots (twigs) is crucial to the study of plant life history strategies because the first-year production of new stems and leaves influences subsequent growth (Westoby *et al.* 2002; Westoby and Wright 2003; Sun *et al.* 2006; Xiang *et al.* 2009; Yan *et al.* 2013). Among the most critical traits of twigs is the relationship between leaf size and number because this relationship has metabolic and mechanical consequences that influence leaf energy balances and carbon uptake at the whole plant level (Kleiman and Aarssen 2007; Ogawa 2008; Milla 2009; Dombroskie and Aarssen 2012; Huang *et al.* 2015).

Prior studies have reported a negative isometric scaling relationship between individual leaf mass and leafing intensity (the number of leaves per stem tissue volume) for twigs (Kleiman and Aarssen 2007; Yang *et al.* 2008; Xiang *et al.* 2010; Huang *et al.* 2015). Kleiman and Aarssen (2007) argued that this trade-off is caused by the fact that small but numerous leaves provide a selective advantage over the course of plant evolution. Therefore, leaf size variation is argued not to be under direct selection, but rather high leafing intensity is a strategy contributing to the maintenance of supernumerary axillary buds (e.g. the more leaves, the more axillary buds) (Kleiman and Aarssen 2007; Dombroskie and Aarssen 2012). However, empirical studies have shown that considerable variation in leaf size and number exists. For example, the scaling exponents governing the leaf size-leafing intensity scaling relationship differ between evergreen and deciduous species (Milla 2009).

The theoretical basis for the leaf size and number trade-off continues to remain elusive. In order to resolve this lack of clarity, we developed a stem-leaf growth hypothesis (SLGH) focusing on the annual stem and leaf growth rates (*G*_L_ and *G*_S_, g year^−1^, respectively) on individual twigs. We tested the predictions of this hypothesis using data collected from 27 families, 45 genera and 64 species floristically representative of three different forest community types (evergreen, mixed and deciduous forest types). We also designed our measurements to correct for a statistical problem that has crept into the literature, i.e. prior studies using mean individual leaf mass (*m*_l_) to test the predictions of any hypothesis about the scaling of leafing intensity are statistically biased because *m*_l_ and leafing intensity are calculated based on leaf number (e.g. Kleiman and Aarssen 2007). Therefore, in the analyses to be presented, the maximum individual leaf size (i.e. leaf mass *M*_max_ and leaf area *A*_max_, reported in g and cm^2^) was used to test the predictions of our hypothesis because *M*_max_ and *A*_max_ obviate the problem of statistical bias and are likely to be representative of maximum leaf growth.

### The SLGH model

Prior studies indicate that total biomass production per individual plant per year scales isometrically with respect to the light-harvesting capacity of an individual (gauged by standing leaf biomass) (Niklas and Enquist 2001), whereas leaf annual growth rates *G*_L_ scale isometrically with respect to stem annual growth rates *G*_S_ (Niklas and Enquist 2002), i.e. *G*_L_ ∞ *G*_S_. It follows therefore that *M*_L_ ∞ *M*_S_, a proportionality that has been empirically demonstrated (e.g. Niklas 2004; Sun *et al.* 2006; Xiang *et al.* 2009). Furthermore, the maximum twig leaf growth in mass must be proportional to the product of maximum individual leaf mass and leaf number (*N*_L_), and be presentative of stem maximum resource support on twigs, i.e. *M*_L_ ∞ *M*_S_ ∞ *M*_max_ × *N*_L._ Thus, it follows that the maximum individual leaf mass should scale negatively and isometrically with respect to leafing intensity, i.e. *M*_max_ ∞ *M*_L_/*N*_L_ ∞ (*N*_L_*/M*_S_)^−1^. Note that stem mass and volume will be proportional to one another provided that the bulk tissue density of stems (ρ) varies little for any particular species, i.e. *M*_S_ = ρ *V*_S_. The hypothesis that annual stem growth (and thus stem mass) scales isometrically with respect to stem cross-sectional area (*A*_S_) as a consequence of hydraulic constraints on water and nutrient transport was tested and supported in previous studies (Fan *et al.* 2017; Sun *et al.* 2019) (i.e. *M*_S_ ∞ *A*_S_). Collectively, therefore, the SLGH predicts *M*_max_ ∞ (*N*_L_*/M*_S_)^−1^, *M*_max_ ∞ (*N*_L_*/V*_S_)^−1^ and *M*_max_ ∞ (*N*_L_*/A*_S_)^−1^. Given that a linear relationship between leaf mass and leaf area exists (Niklas *et al.* 2007; Li *et al.* 2008; Sun *et al.* 2017), our hypothesis also applies to maximum leaf area (*A*_max_) vs. leafing intensity (based on *M*_S_, *V*_S_ or *A*_S_).

## Methods

The predictions of our hypothesis were tested empirically based on data collected from 64 species from three forest types (i.e. an evergreen broad-leaved forest, a conifer and broad-leaved mixed forest and a deciduous broad-leaved forest) growing along an elevational gradient on Wuyi Mountain, China.

### Site description

Data were collected from sample sites at different elevations of the highest mountain in the Huanggang Mountain Range, Wuyi Mountain, in the National Natural Reserve, Jiangxi province, south-eastern of China (27°48′11″N–28°00′35″N, 117°39′30″E–117°55′47″E). The reserve has obvious vertical vegetational zones including *Phyllostachys edulis* forests (below 1400 m a.s.l.), evergreen broad-leaved forests (1000–1400 m a.s.l.), conifer and broad-leaved mixed forests (1400–1800 m a.s.l.), and deciduous broad-leaf forest, and subalpine dwarf forests (1800–1900 m a.s.l.). The reserve is located in the humid warm subtropics in the south-east of China and has a mean annual precipitation of 2583 mm and a mean annual temperature of 14.2 °C. The soil texture is mainly a sandy clay loam (Li *et al.* 2017).

### Sampling

Three forest types were selected along an elevational gradient: (i) an evergreen forest (EF) located at 1319 m a.s.l., with a stand density of 3033 trees per ha, (ii) a mixed forest (MF) located at 1697 m a.s.l., with a stand density of 1133 trees per ha and (iii) a deciduous forest (DF) located at 1818 m a.s.l., with a stand density of 2725 trees per ha. Mean plant height (*H*) was 7.87, 10.56 and 6.94 m in the EF, MF and DF, respectively. Mean trunk diameter at breast height (DBH) was 13.77, 21.39 and 11.47 cm in the EF, MF and DF, respectively.

Three 20 m × 20 m plots were randomly established in each of the three forest communities. A total of 32, 20 and 23 species (including overlapping species) were collected in the EF, MF and DF, respectively. The total number of species in our study was 64, which spanned 45 genera in 27 families. Twigs were defined as terminal current-year shoots, consisting of a single stem and attached leaves (as in Sun *et al.* 2006; Kleiman and Aarssen 2007). For consistency, all of the twigs collected for this study were selected randomly but without apparent leaf area loss, or flowers or fruits. In August 2016, three to five individuals of each species were randomly selected, and five twigs were removed at the perimeter of the crown of each individual. In the case of species with less than three individuals per plot, five twigs from each individual were harvested. All of the leaves on each twig were removed and counted (*N*_L_). Basal twig diameter (*D*, mm) and length (*L*, mm) were measured using a vernier caliper (with an accuracy of 0.1 mm) to determine stem cross-sectional areas (*A*_S_, cm^2^) (Milla 2009) and stem volume (*V*_S_, cm^3^) (i.e. *V*_S_ = *A*_S_ × *L*; a cylindrical geometry was assumed). Stems and leaves were subsequently brought to the laboratory where they were oven-dried at 75 °C to determine total leaf mass (*M*_L_, g) and stem mass (*M*_S_, g). Each leaf of each twig was scanned and its area was calculated using the software Image J; the maximum leaf area was recorded. The area of the largest leaf per twig was multiplied by leaf mass per area (LMA, total leaf mass divided by total leaf area) to estimate the maximum leaf mass per twig. The volume-based leafing intensity (LIV defined as *N*_L_/*V*_S_, cm^3^), mass-based leafing intensity (LIM defined as *N*_L_/*M*_S_, g) and cross-sectional area-based leafing intensity (LIA defined as *N*_L_/*A*_S_, cm^2^) were calculated subsequently. The mean values of leafing intensity and maximum leaf size (*M*_max_ and *A*_max_) for 64 species are provided in **Supporting Information—Table S1**.

### Data analysis

All the data were log_10_-transformed to fit a normal distribution before analysis. Regression analyses showed that the variables of primary interest were log-log linear correlated and conformed to the equation log(*y*_1_) = log(β) + α log(*y*_2_), where β is the normalization constant, α is the scaling exponent and *y*_1_ and *y*_2_ are interdependent variables of interest. When α = 1.0, the scaling relationship is isometric; when α ≠ 1.0, the scaling relationship is allometric (Niklas 1994). Model Type II regression was used to determine the numerical values of β and α using the (standardized major axis (SMA) estimation) package ‘smatr’ version 3.4-3 (Warton *et al.* 2012) in R-3.4.3 software (R Development Core Team 2012). The data from species showing no statistically significant differences in the numerical values of β and α were pooled to determine a common scaling exponent using the SMA package in R-3.4.3 (Warton *et al.* 2006, 2012). The significance level for testing slope heterogeneity was *P* < 0.05 (e.g. slope heterogeneity was rejected when *P* > 0.05). The phylogenetic signals of *M*_max_, *A*_max_, LIV, LIM and LIA were examined using phylogenetically independent contrast (PIC) analysis, which was calculated using the ‘*pic*’ function in the ‘ape’ package in R-3.4.3 software (Paradis *et al.* 2004). The *K*-value was calculated using the ‘*phylosignal*’ function in the ‘picante’ package in R-3.4.3 software (Kembel *et al.* 2010). When *K* > 1, functional traits are said to exhibit a strong phylogenetic signal; *K* < 1 indicates weak phylogenetic signals (Blomberg *et al.* 2003). The twig traits pertaining to our hypothesis showed weak phylogenetic signals across the 64 species **[see****Supporting Information—Table S2****]**; the phylogenetic tree was displayed using iTOL (Letunic and Bork 2016) **[see****Supporting Information—Fig. S1****]**.

## Results

**Figure 1.**
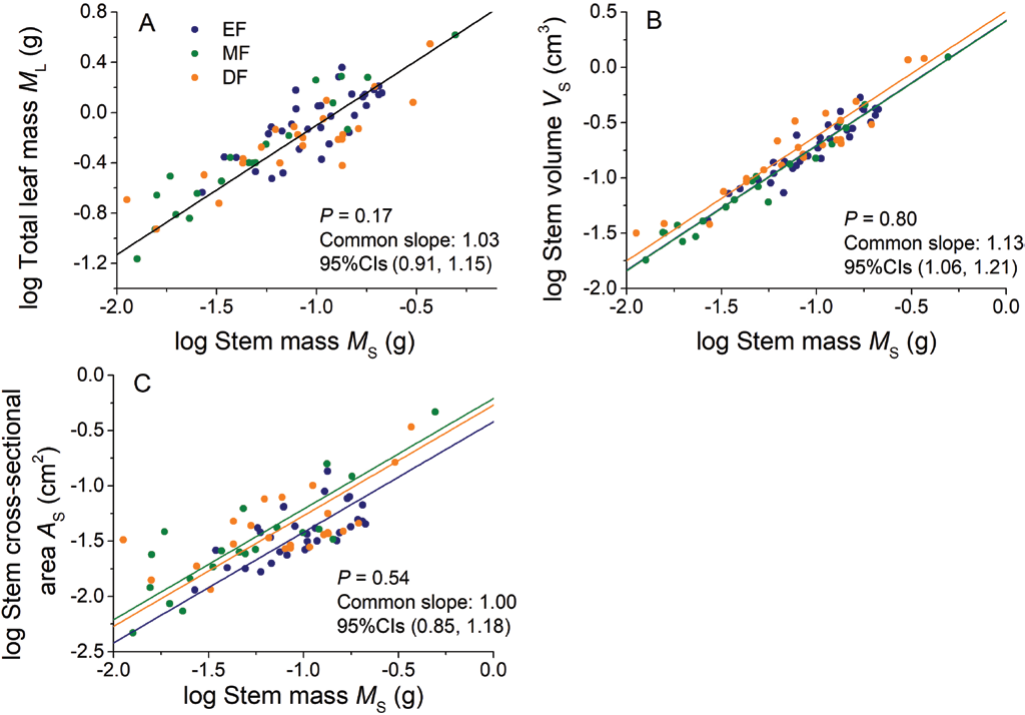
Bivariate plots of total leaf mass vs. stem mass (**A**), Stem volume vs. Stem cross-sectional area (**B**), Stem cross-sectional area vs. Stem mass (**C**). *A*_S_: stem cross-sectional area, the same as below.

Among the three forest types, total leaf mass scaled isometrically with respect to stem mass (Fig. 1A), with a common slope of 1.03 (95 % CI = 0.91 and 1.15, *P* = 0.17). The normalization constants did not differ significantly across the three forest types (Fig. 1A). The scaling exponents for both stem volume vs. stem mass and stem cross-sectional area vs. stem mass did not statistically differ among the three forest types and had a common slope of α =1.13 (95 % CI = 1.06–1.21, *P* = 0.80, Fig. 1B) and α = 1.00 (95 % CI = 0.85−1.18, *P* = 0.54, Fig. 1C), respectively. However, in the preceding two scaling relationships, the scaling exponents of stem volume vs. stem mass were significantly larger than 1.0 (allometric), which is contrary to that observed for the stem cross-sectional area vs. stem mass scaling relationship (Fig. 1B and C).

**Figure 2.**
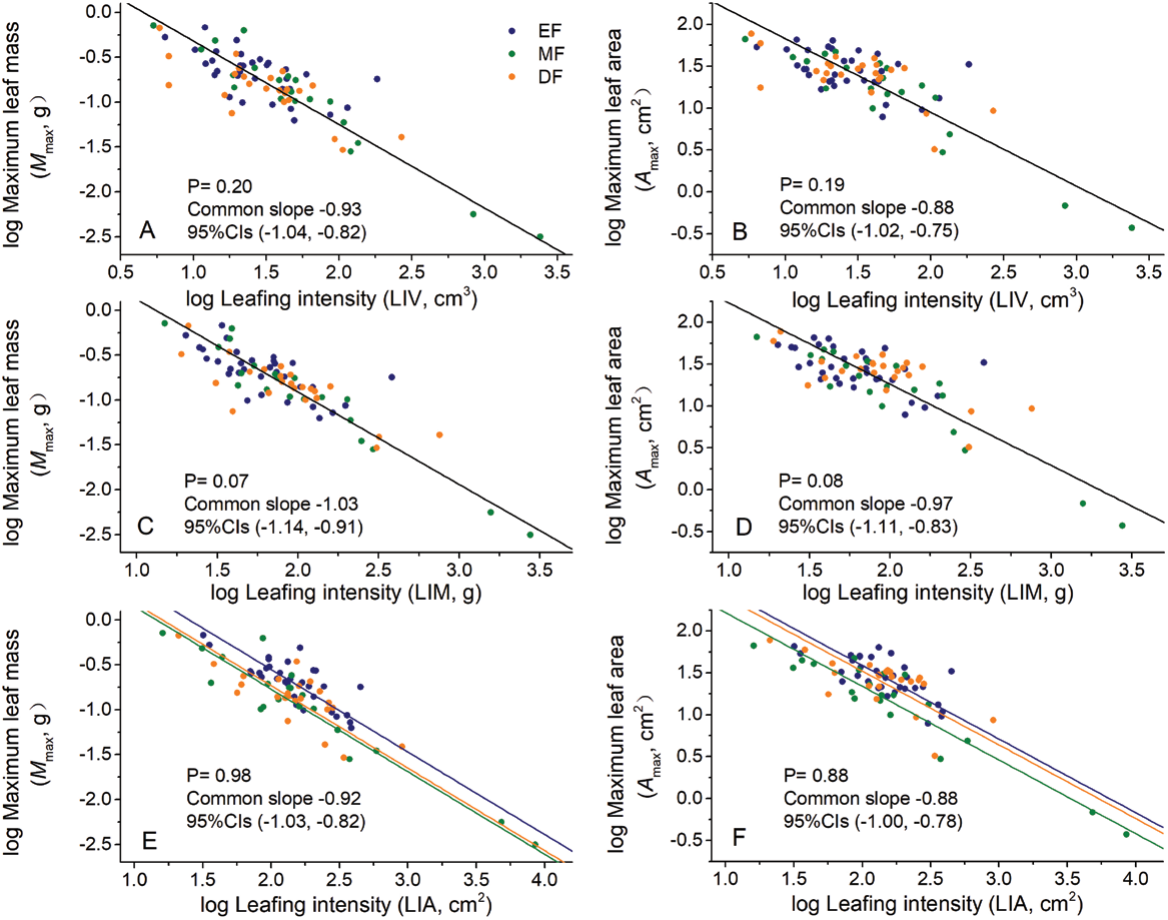
Bivariate plots of the maximum individual leaf size (*M*_max_ or *A*_area_) vs. twig leafing intensity. (**A**) the relationship between *M*_max_ and LIV, (**B**) the relationship between *A*_max_ and LIV, (**C**) the relationship between *M*_max_ and LIM, (**D**) the relationship between *A*_max_ and LIM, (**E**) the relationship between *M*_max_ and LIA, (**F**) the relationship between *A*_max_ and LIA.

Overall, maximum individual leaf size scaled nearly isometrically with respect to the LIV (i.e. α = −0.91 for *M*_max_ and −0.89 for *A*_max_) across the three different forest types (Table 1). The scaling exponents for *M*_max_ vs. LIV were statistically indistinguishable among the three forest types, with a common slope of α = −0.93 (95 % CIs = −1.04 and −0.82, *P* = 0.20). Similarly, the *A*_max_ vs. LIV scaling relationship had a common slope of −0.88 (95 % CIs = −1.02 and −0.75, *P* = 0.19). The numerical values of the normalization constant for the data from the three forest types were not significantly different from one another (i.e. β = 0.61 for *M*_max_ vs. LIV and 2.71 for *A*_max_ vs. LIV) (Fig. 2A and B).

**Table 1.** Summary of regression slopes and *Y*-intercepts (α and log β, respectively) for maximum individual leaf size (*M*_max_ or *A*_max_) vs. leafing intensity (calculated on the basis of stem volume, mass and cross-sectional area: LIV, LIM and LIA, respectively) for the data collected from three different forest types along an elevational gradient. EF: evergreen forest; MF: mixed forest; DF: deciduous forest, *P*_−1.0_ indicated a significant difference test between the slope and 1.0 at 0.05 level. The same as below.

log *y*_1_ vs. log *y*_2_	Forests	*n*	α (95 % CIs)	log β	*r* ^2^	*P*	*P*_−1.0_
*M*_max_ vs. LIV	EF	32	−0.81 (−1.08, −0.61)	0.48	0.40	<0.001	0.15
	MF	20	−1.00 (−1.14, −0.87)	0.75	0.92	<0.001	0.94
	DF	23	−0.79 (−1.06, −0.59)	0.32	0.58	<0.001	0.11
	ALL	75	−0.91 (−1.03, −0.81)	0.58	0.72	<0.001	0.14
*M*_max_ vs. LIM	EF	32	−0.88 (−1.15, −0.68)	0.89	0.47	<0.001	0.35
	MF	20	−1.10 (−1.24, −0.98)	1.28	0.94	<0.001	0.11
	DF	23	−0.83 (−1.07, −0.64)	0.75	0.66	<0.001	0.15
	ALL	75	−1.00 (−1.11, −0.89)	1.08	0.77	<0.001	0.95
*M*_max_ vs. LIA	EF	32	−0.92 (−1.16, −0.73)	1.30	0.60	<0.001	0.48
	MF	20	−0.92 (−1.08, −0.79)	1.08	0.90	<0.001	0.31
	DF	23	−0.89 (−1.18, −0.67)	1.06	0.60	<0.001	0.42
	ALL	75	−0.95 (−1.07, −0.85)	1.26	0.76	<0.001	0.43
*A*_max_ vs. LIV	EF	32	−0.76 (−1.04, −0.55)	2.53	0.23	0.007	0.09
	MF	20	−0.97 (−1.16, −0.81)	2.82	0.87	<0.001	0.76
	DF	23	−0.73 (−1.03, −0.53)	2.48	0.44	<0.001	0.07
	ALL	75	−0.89 (−1.02, −0.77)	2.70	0.64	<0.001	0.09
*A*_max_ vs. LIM	EF	32	−0.82 (−1.20, −0.61)	2.92	0.31	<0.001	0.21
	MF	20	−1.08 (−1.27, −0.91)	3.34	0.89	<0.001	0.35
	DF	23	−0.77 (−1.05, −0.56)	2.88	0.51	<0.001	0.10
	ALL	75	−0.97 (−1.11, −0.85)	3.19	0.66	<0.001	0.64
*A*_max_ vs. LIA	EF	32	−0.86 (−1.12, −0.66)	3.30	0.47	<0.001	0.26
	MF	20	−0.90 (−1.06, −0.77)	3.15	0.89	<0.001	0.20
	DF	23	−0.83 (−1.15, −0.60)	3.17	0.46	<0.001	0.26
	ALL	75	−0.93 (−1.06, −0.81)	3.36	0.68	<0.001	0.26

The scaling exponent for *M*_max_ vs. LIM for each of the three different forest types was not different from −1.0 (all *P*_−1.0_ > 0.05; Table 1). The *M*_max_ vs. LIM and *A*_max_ vs. LIM scaling relationships across all three forest types had a common slope of −1.03 and −0.97, respectively (Fig. 2C and D). Furthermore, there was no significant difference in the numerical values of the normalization constants among the three forest types (i.e. β = 1.15 for *M*_max_ vs. LIM and 3.20 for *A*_max_ vs. LIM, respectively). Both *M*_max_ and *A*_max_ scaled isometrically with respect to LIA among the three forest types, with a common slope of −0.92 and −0.88, respectively. However, the numerical value of the normalization constant for the data from the EF was significantly larger than that of the other two forest types (Fig. 2E and F).

## Discussion

We derived and tested a SLGH that predicts a negative isometric scaling relationship between maximum individual leaf size and leafing intensity (i.e. leaf number per stem volume/stem mass/stem cross-sectional area). The predictions of this hypothesis are shown to be valid using data collected from three different forest types growing along an elevational gradient on Wuyi Mountain, Southeast China. The approach taken here relies on the isometric relationship between stem and leaf growth on current-year stems (twigs), which has been reported by numerous workers analysing growth in different species (e.g. Niklas and Enquist 2002; Niklas 2004; Sun *et al.* 2006; Xiang *et al.* 2009). The key assumption emerging from these studies and incorporated into our hypothesis is that leaf growth is limited by the amount of resources supplied by a stem due to the conservation of nutrients flowing from stems into leaves. This assumption rests on fundamental physical and biological principles as well as empirical observations. For example, the ‘pipe model’ of Shinozaki *et al.* (1964a, b) stipulates that the cross-sectional area of the vascular tissues in twigs (stems that have yet to undergo secondary growth) should be proportional to leaf surface area and thus leaf biomass (see also Niklas 1992, 1994; Niklas and Spatz 2012). In this context, using data from the twigs of 69 temperate tree species, Brouat *et al.* (1998) report a strong isometric relationship between stem cross-sectional area and leaf biomass. Although some studies show that there is a scaling relationship between leaf area and the cross-sectional area of sapwood, this relationship changes systematically as tree height increases and only becomes stable when growth in height ceases (McDowell *et al.* 2002; Mokany *et al.* 2003). Nevertheless, leaf annual growth rates are reported to scale isometrically with stem annual growth rates across woody and non-woody species (e.g. Niklas and Enquist 2002, 2004; Sun *et al.* 2006; Yang *et al.* 2008). Further, using data for global forest biomass and growth compiled by Cannell (1982), Niklas and Enquist (2003) have shown, both theoretically and empirically, that annual biomass production scales isometrically with respect to leaf, stem and root growth rates. Our results also show that total leaf mass scales isometrically with respect to stem mass on current-year twigs across the three forests (Fig. 1A). Thus, there is reliable and sufficient evidence that the hypothesis that annual growth rates across all three plant organ types scale isometrically is valid. It is reasonable to suppose that the resources hydraulically supplied by twigs depend on stem cross-sectional area and thus cross-sectional area is correlated with stem and leaf growth rates, a supposition that has been demonstrated empirically (Brodribb and Feild 2000; Normand *et al.* 2008; Fan *et al.* 2017). Previously, we argued that stem diameter (thus stem cross-sectional area) is the primary constraint on the total leaf biomass in twigs (Sun *et al.* 2019). Our data also show that stem cross-sectional area scales isometrically with respect to stem mass (Fig. 1C). Therefore, the leaf mass vs. leaf number trade-off should be dictated by the isometric growth rates of stems and leaves (Kleiman and Aarssen 2007).

The data gathered over the course of this study come from three different kinds of forest communities. Yet, the data from each forest type comply with the SLGH, particularly the prediction that the scaling between maximum individual leaf size and leafing intensity will manifest a negative isometric relationship (Table 1; Fig. 2). Our data and the SLGH hypothesis are also consistent with prior studies. For example, Kleiman and Aarssen (2007) as well as others (Milla 2009; Whitman and Aarssen 2010; Xiang *et al.* 2010; Yan *et al.* 2012, 2013; Huang *et al.* 2015) report a negative isometric relationship between leaf intensity and mean individual leaf mass. However, the interpretation of the results reported in these and some other studies is problematic because of a statistical problem, i.e. average leaf mass should not be calculated by dividing total leaf mass by total leaf number when leafing intensity is calculated using the total leaf number per stem. To the best of our knowledge, the data presented here are the first to avoid this statistical problem and the first to be embedded within and driven by a simple biophysical model.

Specifically, our analyses used the maximum individual leaf mass and maximum individual leaf area per twig, which was calculated without using leaf number in any way. This approach directly establishes the upper limit of leaf size that can be supported by the ability of a stem to provide resources. It is important to note that the numerical values of the normalization constant for *M*_max_ vs. LIA and *A*_max_ vs. LIA differ among the three forest types, which indicates that, for any given stem cross-sectional area, the maximum leaf growth rates differ among the three forest types (Fig. 2E and F). Compared with stem mass or volume, leafing intensity based on stem cross-sectional area (a gauge of resource supply) provides quantitative evidence that the scaling for leaf size vs. leafing intensity differs among different functional groups (Milla 2009). Indeed, compared with the other two forests, the twigs harvested from the evergreen forest support a greater maximum leaf mass or area per unit LIA (Fig. 2E and F), i.e. the evergreen forest has a higher leafing intensity (perhaps to maximize leaf growth), but is constrained by a lower conductive capacity. In contrast, the deciduous forest has a higher stem volume (but lower wood density, Fig. 1B) for the equivalent stem mass of the evergreen forest, which has a larger stem cross-sectional area (perhaps to maximize the resource supply provided by a lower leafing intensity). In addition, the data indicate that *M*_max_ scales one-to-one (α = 1.0) with mean individual leaf mass (data not published), which indicates that maximum individual leaf mass makes a larger contribution to mean individual leaf mass, as well as to the total leaf mass per twig. Thus, maximum individual leaf mass may be a more reliable measure of leaf growth rates and resource utilization efficiency compared to mean leaf mass.

Our data also reveal that, for any given maximum individual leaf size, trees living at higher altitudes produce twigs with fewer leaves compared to trees living at lower altitudes (Fig. 2E and F). We offer two possible explanations for this finding. First, trees living at higher altitudes experience more frequent exposure to more intense wind speeds such that a reduction in the number of leaves provides an adaptive response (because it reduces wind-induced drag forces on stems) (Li *et al.* 2008), while providing more resources to support the total investment in leaf mass (Pan *et al.* 2013; Erasto *et al.* 2014). A second plausible explanation is that lower leafing intensity at higher altitudes might help to decrease the possibility of embolisms at low temperatures. Because low temperatures usually result in a low transporting efficiency (Mayr *et al.* 2006), species from high altitudes support more investments in transport tissues compared to species growing at lower altitudes. For example, some plants in high mountains have less lamina area with respect to a given petiole investment (Li *et al.* 2008). Clearly, these are not mutually exclusive explanations, because both factors (i.e. drag forces at high wind speeds and embolism at low temperatures) are correlated positively with increasing altitude.

Importantly, the isometric scaling relationships reported here can be extended theoretically to the whole plant level. Our results indicate that the maximum total leaf size per twig scales isometrically with respect to stem cross-sectional area at the level of individual twigs, i.e. *A*_S_ ∞ *M*_max_ × *N*_L_ ∞ *M*_L_. Because this relationship should hold for every twig within the canopy of a woody plant, leaf mass at the level of the whole canopy should scale as the sum of the total cross-sectional area of all the twigs in the canopy. Provided that the sum of the cross-sectional areas of all the twigs scales one-to-one with tree trunk diameter *D* as *A*_S_ ∞ *D*^2^ (as reported by Bond-lamberty *et al.* 2002; Niklas 2004), leaf mass at the whole tree level should scale isometrically with respect to the cross-sectional area of the trunk. It is noteworthy that this prediction is supported by a number of theoretical and empirical studies (e.g. West *et al.* 1997, 1999; Enquist *et al.* 1998; Enquist and Niklas 2002; Brown *et al.* 2004). Clearly, however, this expectation requires future empirical exploration. We offer it here simply to emphasize that the SLGH hypothesis (and our data) is consistent with previous theoretical and empirical studies.

An important caveat regarding the ‘negative isometric’ trade-off between maximum individual leaf mass and leafing intensity is the assumption that an isometric relationship between leaf and stem mass exists for twigs. In a more general context, if the scaling exponent between *M*_L_ and *M*_S_ is α ≠ 1.0, our leaf-stem growth hypothesis shows that the scaling for the trade-off between maximum leaf size and leafing intensity is governed by −α. Indeed, some studies have shown that the scaling exponents governing the leaf size vs. number scaling relationship differ significantly among diverse communities (e.g. Yang *et al.* 2008; Milla 2009), perhaps because stem biomass allocation and tissue density vary across community types (Enquist and Niklas 2001; Ogawa 2008). Therefore, it is clear that a universal exponent does not exist for all plant communities.

## Conclusions

The SLGH hypothesis successfully predicts the trade-off between maximum individual leaf size and leaf number observed for current-year shoots (twigs), and it is here validated using data collected for three different kinds of forest communities. This hypothesis provides a theoretical basis for understanding the trade-off between maximum leaf growth and number on twigs, regardless of the numerical value of the scaling exponent (i.e. when α = 1.0 or α ≠ 1.0). Future work is required to further test our hypothesis, and for comparing data from different plant families and species groups.

## Supporting Information

The following additional information is available in the online version of this article—

**Table S1.** Trait means for the 64 species at the twig level along an elevational gradient in Wuyi Mountain.

**Table S2.** Phylogenetic signal of twig functional traits in three forests.

**Figure S1.** Phylogenetic tree of the whole compilation data set (64 species). The topology displayed was obtained from the maximally resolved seed plant tree available in Phylomatic (http://www.phylodiversity.net/phylomatic). Numbers in parentheses are number of species overlapped by each species across the three forests data set.

## Sources of Funding

This work was supported by the National Natural Science Foundation of China (31722007), the National Key Research and Development Program of China (2017YFC0505400) and the Fujian Natural Science Funds for Distinguished Young Scholars (2018J07003).

## Contributions by the Authors

Conceived and designed the experiments: J.S., M.T.W., D.L.C. Performed the experiments: J.S., M.L., Q.L.Z., M.L. Analysed the data: J.S., M.T.W., M.L. Developed the hypothesis and wrote the paper: J.S., M.T.W., K.J.N., D.L.C. All authors read and commented on this manuscript.

## Supplementary Material

plz063_suppl_Supporting_Information
