# Supplementary material for: Stem and leaf growth rates define the leaf size vs. number trade-off
Source: AoB Plants. 2019 Nov 19;11(6):plz063. doi: 10.1093/aobpla/plz063 (PMC6863467; doi:10.1093/aobpla/plz063)

Supporting Information

Table S1. Trait means for the 64 species at the twig level along an elevational gradient in Wuyi Mountain.

Forest types	Species	M_{max} (g)	A_{max} (cm^2)	LIV	LIM	LIA
Evergreen forest	*Fagus longipetiolata*	0.366	49.223	14.354	25.713	96.140
	Castanea henryi	0.342	63.622	21.228	41.660	132.472
	Lithocarpus iteaphyllus	0.273	28.055	32.804	70.806	212.927
	Pterostyrax corymbosus	0.231	44.626	43.264	67.278	85.166
	Cerasus pseudocerasus	0.205	33.371	59.683	73.826	127.427
	Adinandra millettii	0.528	53.840	6.391	20.162	35.330
	Ilex chinensis	0.221	24.792	14.577	38.226	110.734
	Cyclobalanopsis glauca	0.183	21.187	26.529	59.135	189.474
	Castanopsis eyrei	0.491	54.076	19.786	36.191	162.952
	Ilex wilsonii	0.072	9.536	87.054	165.016	380.193
	Rhododendron ovatum	0.087	13.174	114.460	197.468	359.873
	Symplocos sumuntia	0.288	29.174	13.490	27.147	92.668
	Corylopsis sinensis	0.140	27.582	45.961	123.325	148.183
	Carpinus viminea	0.099	18.448	21.767	48.683	172.769
	Cornus controversa	0.257	51.163	21.065	44.500	82.604
	Cyclobalanopsis gracilis	0.181	20.817	43.825	82.317	244.290
	Ternstroemia gymnanthera	0.678	65.441	11.986	33.768	31.991
	Schima superba	0.300	36.826	28.728	70.733	97.333
	Ilex ficoidea	0.257	49.008	31.945	92.970	116.614
	Rhododendron latoucheae	0.196	20.844	20.170	37.249	131.732
	Erythroxylum sinensis	0.063	10.921	49.270	136.498	387.370
	Itea oblonga	0.201	29.051	13.999	43.291	169.078
	Castanopsis fargesii	0.140	20.456	40.917	103.730	205.835
	Albizia kalkora	0.180	32.971	183.489	382.367	449.838
	Cyclobalanopsis multinervis	0.093	21.375	34.966	85.969	278.337
	Illicium minwanense	0.268	32.400	12.150	31.932	70.494
	Castanopsis carlesii	0.274	36.474	25.382	51.946	204.139
	Ilex pedunculosa	0.084	7.813	46.673	124.266	302.032
	Litsea rotundifolia	0.253	24.897	22.019	72.046	71.761
	Cinnamomum pauciflorum	0.384	50.164	10.257	24.482	96.205
	Symplocos wikstroemiifolia	0.113	16.681	17.714	59.639	153.294
	Cyclobalanopsis myrsinifolia	0.218	21.342	20.938	46.290	137.981
Mixed forest	*Illicium minwanense*	0.388	40.367	11.317	32.213	44.151
	Rhododendron simiarum	0.484	36.091	14.140	37.896	31.385
	Tsuga chinensis	0.003	0.372	2414.352	2757.869	8554.307

	Litsea pungens	0.146	17.175	19.054	42.476	170.298
	Eurya Saxicola	0.028	2.973	120.539	292.880	374.531
	Rhododendron latoucheae	0.176	14.708	50.306	74.944	135.540
	Cyclobalanopsis multinervis	0.241	30.232	26.459	53.382	141.141
	Lithocarpus glaber	0.630	47.119	22.218	38.955	87.413
	Acer palmatum	0.130	22.851	47.153	64.248	113.352
	Viburnum setigerum	0.199	44.491	18.801	44.596	36.430
	Eurya brevistyla	0.109	9.932	40.055	89.373	161.084
	Taxus chinensis	0.006	0.684	837.140	1574.888	4847.228
	Halesia macgregorii	0.102	29.992	49.992	109.199	205.888
	Camellia fraternal	0.035	4.860	135.586	248.717	590.601
	Cornus hongkongensis	0.107	15.507	63.349	141.358	87.964
	Sorbus alnifolia	0.102	18.539	87.313	202.724	84.146
	Symplocos sumuntia	0.175	17.100	38.933	95.593	139.105
	Rhododendron fortunei	0.715	66.778	5.318	14.907	16.161
	Carpinus viminea	0.059	13.273	107.964	212.394	306.815
	Acer elegantulum	0.194	34.101	44.759	72.192	94.930
Deciduous forest	*Illicium minwanense*	0.237	31.684	20.581	78.438	62.915
	Meliosma cuneifolia	0.160	24.925	24.296	79.507	227.916
	Agapetes lacei	0.041	9.276	269.032	758.517	248.531
	Alpinia japonica	0.101	25.881	41.964	111.724	258.361
	Symplocos paniculata	0.133	28.421	53.496	120.677	164.843
	Symplocos urceolaris	0.154	17.498	6.765	30.995	56.522
	Clethra barbinervis	0.141	29.505	31.748	159.414	133.610
	Padus avium	0.184	32.295	33.919	78.202	159.965
	Viburnum sympodiale	0.323	59.379	6.805	18.933	38.068
	Lindera erythrocarpa	0.105	23.176	45.127	130.977	282.713
	Ilex tsoii	0.075	21.614	18.432	39.462	132.881
	Rhododendron fortunei	0.670	77.447	5.854	20.934	21.157
	Lindera obtusiloba	0.125	32.622	42.543	127.211	150.025
	Magnolia amoena	0.191	41.040	22.252	89.934	60.730
	Cyclobalanopsis multinervis	0.343	33.918	19.656	37.563	153.647
	Photinia beauverdiana	0.220	39.158	41.105	61.793	113.775
	Acer nikoense	0.119	27.492	16.407	65.811	263.952
	Eurya brevistyla	0.135	15.344	39.105	95.110	127.769
	Acer palmatum	0.152	29.983	66.017	90.813	132.435
	Eurya Saxicola	0.029	3.219	106.070	307.802	339.896
	Zanthoxylum simulans	0.039	8.595	93.556	318.415	910.008
	Fraxinus chinensis	0.206	26.111	19.293	50.377	193.418
	Stewartia gemmata	0.139	21.954	44.053	106.738	112.091

Notes: Maximum individual leaf mass, M_{max}; Maximum individual leaf area, A_{max}; Leafing intensity based on stem volume, LIV; Leafing intensity based on stem mass, LIM; Leafing intensity based on stem cross-sectional area, LIA. The same as below.

Table S2 Phylogenetic signal of twig functional traits in three forests.

	K	P
LIV	0.43	0.08
LIM	0.32	0.21
LIA	0.68	0.01
M_{max}	0.40	0.24
A_{max}	0.52	0.17

Figure S1. Phylogenetic tree of the whole compilation data set (64 species). The topology displayed was obtained from the maximally resolved seed plant tree available in Phylomatic (http://www.phylodiversity.net/phylomatic). Numbers in parentheses are number of species overlapped by each species across the three forests data set.

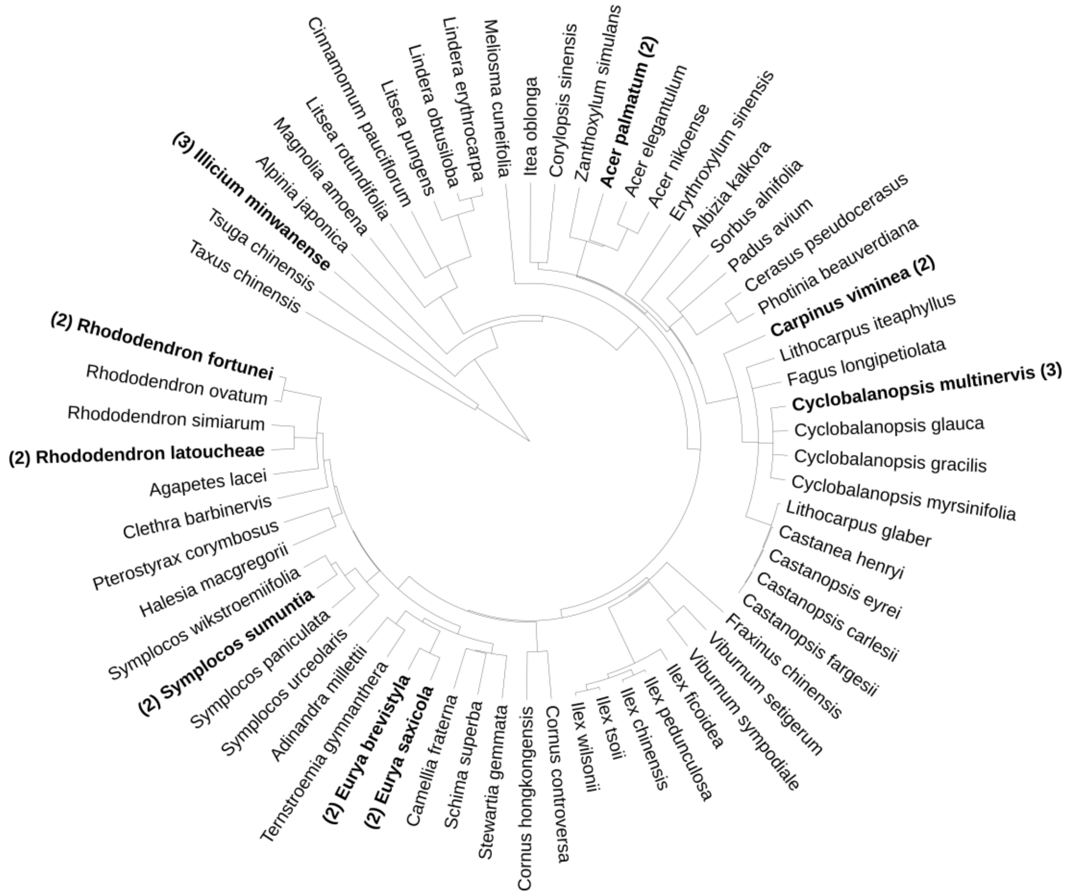

Supplement: plz063_suppl_Supporting_Information [file plz063_suppl_supporting_information.pdf]
